# Supplementary material for: Asymmetric vibrations in the organ of Corti by outer hair cells measured from excised gerbil cochlea
Source: Commun Biol. 2024 May 18;7:600. doi: 10.1038/s42003-024-06293-4 (PMC11102476; doi:10.1038/s42003-024-06293-4)
Supplement: Supplementary file 2 — Supplementary Information [file 42003_2024_6293_MOESM2_ESM.pdf]

## Supplementary Information

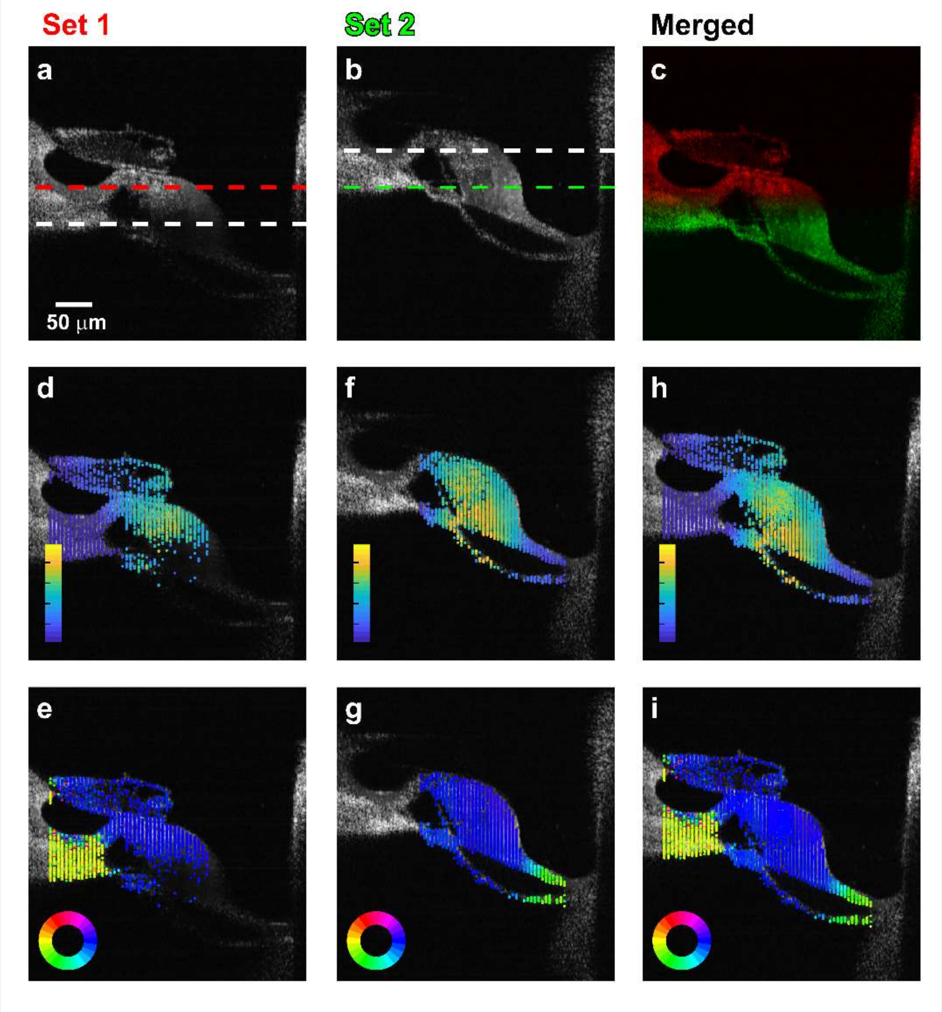


**Supplementary Figure 1**. **Merging two measurements at different focal depths.** (**a**) B-scan image when the focal plane was near the top of the OoC (red broken line). (**b**) B-scan image when the focal plane was near the bottom of the OoC. (**c**) Two images of (a, b) were merged. (**d**, **e**) Measured vibration amplitude and phase when the focal plane was at the OoC top. (**f**, **g**) Measured vibration amplitude and phase when the focal plane was at the OoC bottom. (**h, i**) Merged data have rich signals throughout the depth of OoC. All B-scan images share the same scale as panel a.  Data sets used: M0928 of year 2022.

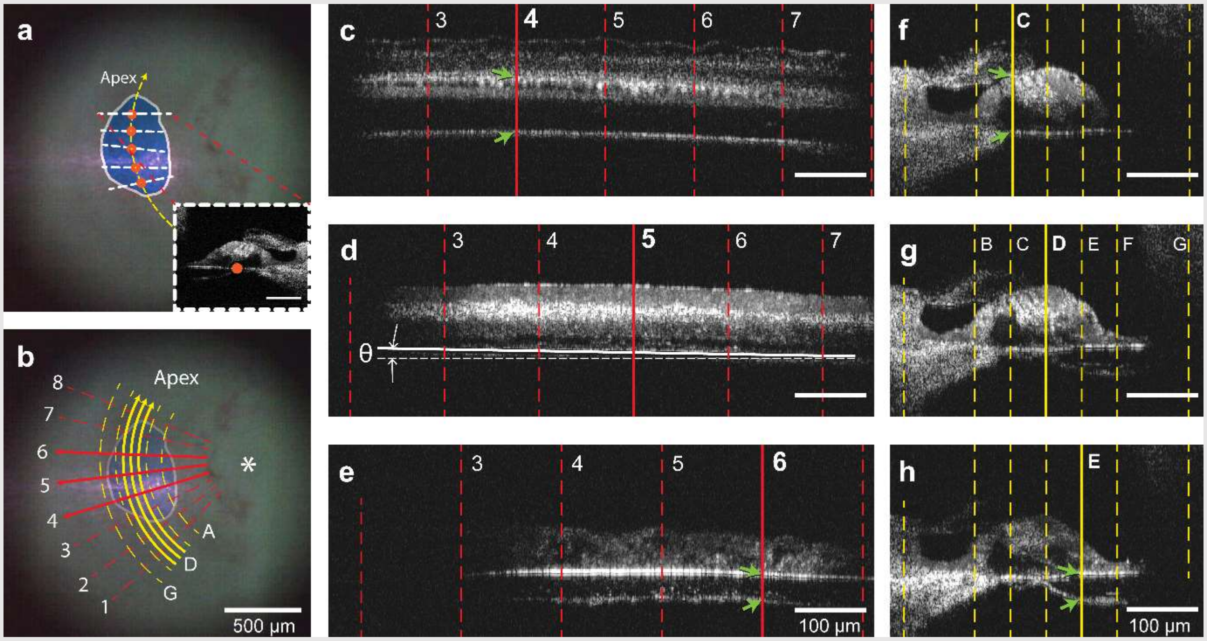


**Supplementary Figure 2**. **Determining the cochlear axes** (**a**) The camera image. The shaded blue region indicates the opening after removing the inter-scala bone. The broken white lines denote the initial B-scan lines. One of the scans is shown in the inset figure. On each scan, the location of a consistent anatomical point is marked with an orange dot (usually the root of the outer pillar cell). The yellow arc indicates the line of curve-fit along the user-identified anatomical points. (**b**) The cochlear coil is assumed to be piecewise-circular. The white asterisk at the modiolus is the center of the coil piece. The outer hair cells are along the thick yellow arcs (the radial points C, D and E). Defined radial sections (straight red lines) are normal to the arcs. (**c**) A longitudinal section along the radial point C. (**d**) A longitudinal section along the radial point D. (**e**) A longitudinal section along the radial point E. (**f**) A radial section along the scan line #4. (**g**) A radial section along the scan line #5. (**h**) A radial section along the scan line #6. The green arrows in panels c and d indicate the same anatomical sites. The same applies to the green arrows in panels e and h.

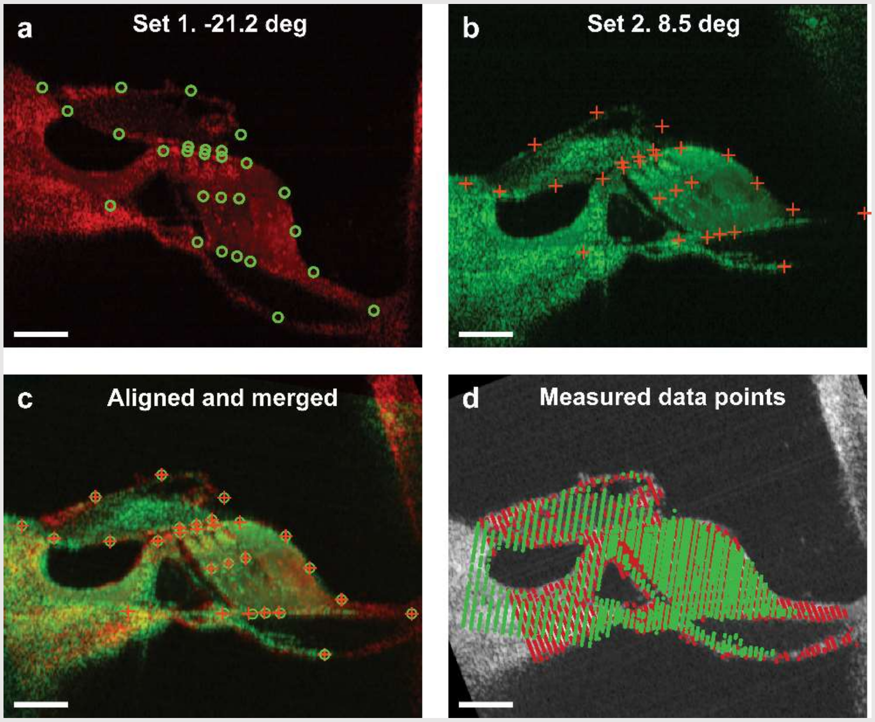


**Supplementary Figure 3**. **Alignment between measurements at two orientation angles.** (**a**) Identified anatomical points at the orientation angle of -21.2 degrees. Twenty-seven points were indicated with green circles. (**b**) We tried to find the same anatomical points as set 1 at the orientation angle of 8.5 degrees. (**c**) The two measurements (images as well) were aligned. The nodes corresponding to the outer hair cells match well. In contrast, less confident nodes, such as the roots of Deiters cells, show some errors. (**d**) OCT vibrometry data points from the two orientation angles were presented together. Some structures have good signals in one orientation but not so in the other orientation. In this example, the outer pillar cell was identified clearly in set 2, but it is not visible in set 1. The four plots are on the same scale and the scale bars indicate 50 µm. Data sets used: M0928 of year 2022.

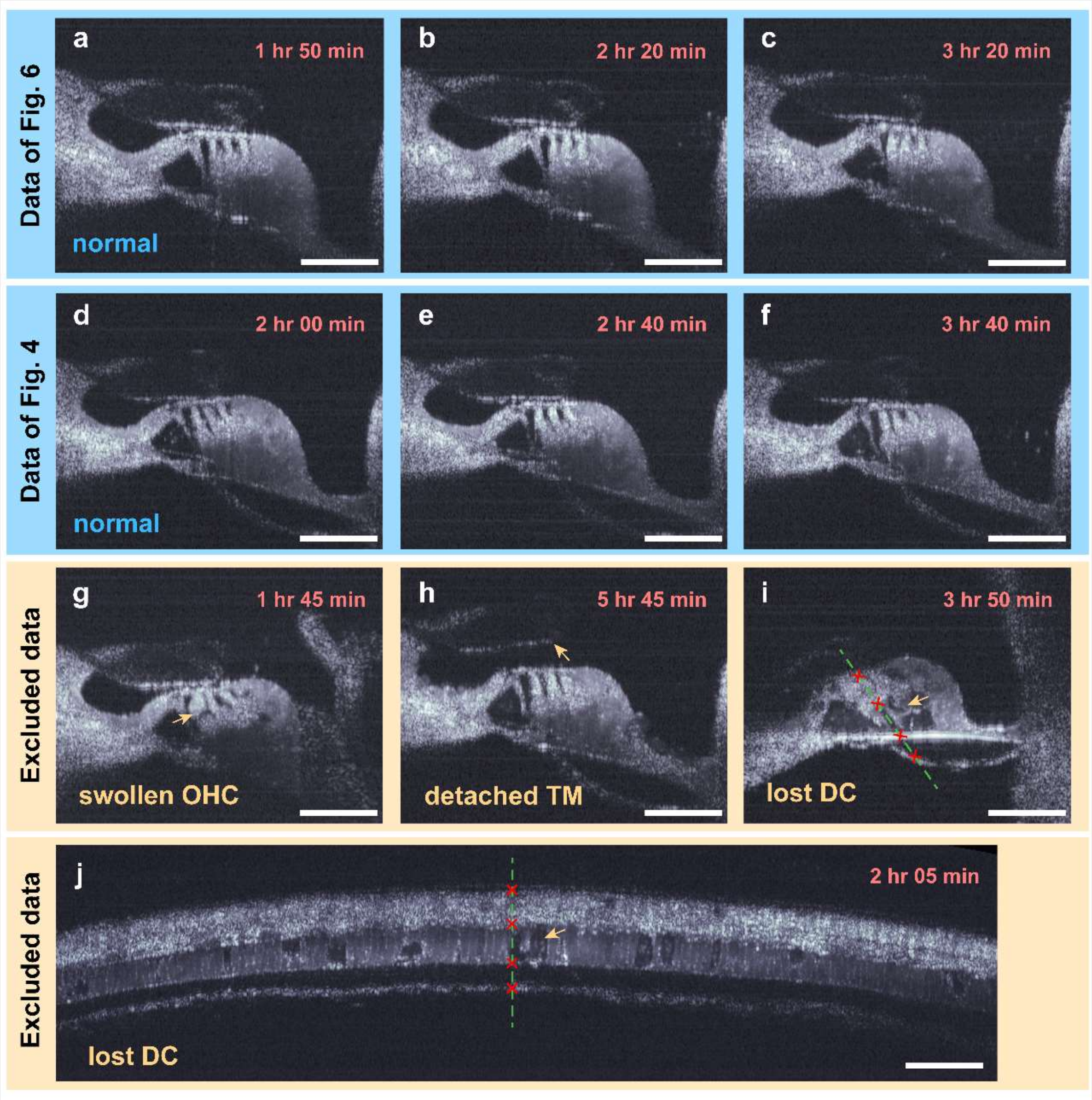


**Supplementary Figure 4**. **The structural integrity of experimental preparations.** B-scan images are from four sample cochleae. The B-scan images inform the structural integrity of our preparations. (**a-c**) B-scan images of Fig. 6 preparation at different time points from animal death (indicated in the top right corner of each panel). (**d-f**) B-scan images of Fig. 4 preparation at three time points. (**g-i**) Three examples of data excluded due to abnormal morphology: swollen outer hair cells (g), detached tectorial membrane (h), and lost Deiters' cells (i). (**j**) longitudinal scan of the same cochlea as (i). The green broken lines indicate where the corresponding radial/longitudinal sections were taken. These negative examples were acquired while refining current experimental protocols before obtaining the data set used for this study. The data in this paper were obtained between June 2022 and January 2023. The last observation of badly swollen outer hair cells was in April 2022. Lost Deiters' cells are rare (they have not been observed since 2021). Meanwhile, delaminated tectorial membranes were observed during the experimental period of this study. The scale bars are 100 µm. Detached TM was observed in 4 out of 26 measurements. Scale bars: 100 µm. Data sets used: 0822, 0826, 0304 of year 2022, 0919 of year 2021.

# Captions for Supplementary Movies

**Supplementary Movie 1. 2D motion**

Two-D motion due to M-stim is followed by E-stim motion. For respective stimulation cases, responses at four stimulating frequencies between 1 and 4 kHz are shown. The M-stim responses were from the 'TM-up' preparation, while the E-stim responses were from the 'BM-up' preparation. The amplitude of motion and the time scale were exaggerated for illustration (approximately a thousand times greater or slower). This movie corresponds to Fig. 2. The data sets used were M0928 and E1220.

Link to FigShare: https://figshare.com/s/aeeb39947158b682e790 (DOI: 10.6084/m9.figshare.23710605)

**Supplementary Movie 2. Basilar membrane (individual)**

The vibrating pattern of the basilar membrane due to M-stim and E-stim. For respective stimulation cases, responses to five stimulating frequencies between 1 and 5 kHz are shown. This movie corresponds to Fig. 3. The data sets used were M1220 and E1220.

Link to FigShare: https://figshare.com/s/0814a1ceb7db0047645f (DOI: 10.6084/m9.figshare.23710587)

**Supplementary Movie 3. Basilar membrane (together)**

Basilar membrane vibrating patterns at four frequencies (1, 2, 4, and 8 kHz) are shown together. The time scale was normalized so that the animated periods would be the same despite frequencies. Note the mode-shift of the M-stim case: primary mode at 1 and 2 kHz; transition at 4 kHz; higher-order mode at 8 kHz. For E-stim, regardless of stimulating frequency, the basilar membrane vibrated like the higher-order mode of M-stim. This movie corresponds to Fig. 4. The data sets used were M1220 and E1220.

Link to FigShare: https://figshare.com/s/9697ae26bbd3a2dedce4 (DOI: 10.6084/m9.figshare.23710611)

**Supplementary Movie 4. Reticular lamina**

Reticular lamina vibrating patterns due to M-stim and E-stim. For M-stim, the reticular lamina moved like a rigid bar. In contrast, the reticular lamina deflected for E-stim. Despite the difference between stimulation types, the vibrating patterns remained similar over the 3-octave frequency range (shown a 2-octave range). This movie corresponds to Fig. 5. The data sets used were M1220 and E1220.

Link to FigShare: https://figshare.com/s/e6f4cfe78b26e120ac4c (DOI: 10.6084/m9.figshare.23710614)

**Supplementary Movie 5. Outer pillar cell**

Outer pillar cell vibrating patterns due to M-stim and E-stim. For M-stim, the outer pillar cell pivots about the inner pillar cell root. For E-stim, the outer pillar cell deflects with minimal motion at its joint with the basilar membrane. This movie corresponds to Fig. 6. The data sets used were M0928 and E0621.

Link to FigShare: https://figshare.com/s/c9445e689c8ec07428ec (DOI: 10.6084/m9.figshare.23710599)

**Supplementary Movie 6. Summary**

For M-stim, the Corti scaffold (the tunnel of Corti and the reticular lamina) acted like a stiff truss that transmitted the acoustic force to the upper part of the OoC. In contrast, for E-stim, the Corti scaffold deflected like an elastic frame clamped at its edges. Note that the motion was asymmetrical, so the top side of OoC vibrated much more than the bottom side of OoC. The Corti frame helps to asymmetrical power delivery from the outer hair cells. The Deiters cells of the E-stim case are in gray to indicate that their motion was not measured but that they were drawn to assist the eyes. This movie corresponds to Fig. 7. The data sets used were M0928 and E0621.

Link to FigShare: https://figshare.com/s/1fe648b485473a13d0de (DOI: 10.6084/m9.figshare.23710590)
